# A Systematic Review of Taste Differences Among People With Eating Disorders

**DOI:** 10.1177/1099800419872824

**Published:** 2019-09-04

**Authors:** Ariana M. Chao, Abhrarup Roy, Alexis T. Franks, Paule V. Joseph

**Affiliations:** 1 Department of Biobehavioral Health Sciences, The University of Pennsylvania School of Nursing, Philadelphia, PA, USA; 2 Center for Weight and Eating Disorders, Department of Psychiatry, Perelman School of Medicine, University of Pennsylvania, Philadelphia, PA, USA; 3 Sensory Science & Metabolism Unit, Biobehavioral Branch, Division of Intramural Research, Department of Health and Human Services, National Institutes of Health, Bethesda, MD, USA

**Keywords:** anorexia nervosa, bulimia nervosa, binge eating disorder, eating disorder, taste, neuroimaging, fMRI

## Abstract

**Background:**

Eating disorders are a significant cause of morbidity and mortality. The etiology and maintenance of eating-disorder symptoms are not well understood. Evidence suggests that there may be gustatory alterations in patients with eating disorders.

**Objective:**

This article systematically reviews research assessing gustatory differences in patients with anorexia nervosa (AN), bulimia nervosa (BN), and binge eating disorder (BED).

**Method:**

A systematic review was performed, following Preferred Reporting Items for Systematic Reviews and Meta-Analyses guidelines, examining taste and eating disorders. We reviewed electronic databases and identified 1,490 peer-reviewed English-language studies. Of these, 49 met inclusion criteria.

**Results:**

Studies employed psychophysical measures (*n* = 27), self-reported questionnaires (*n* = 5), and neuroimaging techniques (i.e., electroencephalography, functional magnetic resonance imaging; *n* = 17). Psychophysical studies showed that individuals with BN, in general, had greater preference for sweetness than healthy controls, and those with AN had a greater aversion for fat than controls. In neuroimaging studies, findings suggested that predictable administration of sweet-taste stimuli was associated with reduced activation in taste-reward regions of the brain among individuals with AN (e.g., insula, ventral, and dorsal striatum) but increased activation in BN and BED.

**Discussion:**

To our knowledge, this systematic review is the first to synthesize literature on taste differences in AN, BN, and BED. The inconsistency and variability in methods used across studies increased difficulties in comparing studies and disease processes. Further studies with well-defined population parameters are warranted to better understand how taste varies in patients with eating disorders.

Eating disorders are among the most common psychiatric disorders in the United States and are a significant cause of morbidity and mortality in young adults (Hudson, Hiripi, Pope, & Kessler, 2007; Westmoreland, Krantz, & Mehler, 2016). The *Diagnostic and Statistical Manual of Mental Disorders* includes three major eating-disorder classifications: anorexia nervosa (AN), bulimia nervosa (BN), and binge eating disorder (BED; American Psychiatric Association [APA], 2013). AN is a disorder characterized by restriction of energy intake relative to that required for survival, severe anxiety or fear of gaining weight, and altered perception of body image (APA, 2013). In the United States, this disorder affects 0.9−2.0% of females and 0.1−0.3% of males (Keski-Rahkonen et al., 2007; Stice, Marti, Shaw, & Jaconis, 2009). Symptoms of BN include repeated incidents of binge eating or ingestion of a large amount of food in a discrete time period while feeling a sense of loss of control over eating followed by inappropriate compensatory mechanisms to avoid weight gain (e.g., self-induced vomiting and excessive exercise). The prevalence of BN in the United States is 1.1−4.6% among females and 0.1−0.5% among males (Favaro, Caregaro, Tenconi, Bosello, & Santonastaso, 2009; Hoek & van Hoeken, 2003; Hudson et al., 2007). BED, the most common eating disorder, affects 0.2−3.5% of females and 0.9−2.0% of males (Hudson et al., 2007; Stice et al., 2009) and is characterized by repeated binge eating without the associated compensatory mechanisms seen in BN. The etiology and maintenance of eating-disorder symptoms are not well understood. Evidence suggests, however, that there may be gustatory alterations in patients with eating disorders.

The sense of taste is linked to brain-reward processes that may drive eating behavior, food selection, consumption, and preference (Drewnowski, Krahn, Demitrack, Nairn, & Gosnell, 1992; Garcia-Bailo, Toguri, Eny, & El-Sohemy, 2009; van Dongen, van den Berg, Vink, Kok, & de Graaf, 2012). Taste studies often assess taste ability, quality and intensity, and pleasantness/unpleasantness. In behavioral studies, researchers frequently measure taste using psychophysical methods that measure the associations between physical stimuli and perceptions. Taste perception can also be assessed using neuroimaging techniques that examine neural correlates of the taste sensory system (Hoogeveen, Dalenberg, Renken, ter Horst, & Lorist, 2015). Differences in taste may be explained by differences in brain structure (Fjell et al., 2006) or function (Hoogeveen et al., 2015). Upon stimulation of taste receptor cells in the tongue, gustatory information is transmitted to the brain via specialized sensory branches of cranial nerves. These transmitted signals code for basic taste quality and concentration (Di Lorenzo & Victor, 2003) to the rostral division of nucleus tractus solitarius on the medulla (Simon, de Araujo, Gutierrez, & Nicolelis, 2006). Research has implicated the amygdala and lateral hypothalamus in taste processing (Anderson & Sobel, 2003; Small et al., 2003).

Treatment options for eating disorders remain limited in part because factors underlying the etiology and maintenance of these disorders are not well understood. Despite the possible importance of taste in the psychopathology of eating disorders and as a possible clinical target for treatment, to date, no systematic reviews assessing taste (measured psychophysically or by neuroimaging) have been conducted across eating disorders. Thus, the purpose of this article is to review research assessing taste differences in people with AN, BN, or BED. Highlighting potential similarities and differences in taste perception across these groups may provide insight into shared mechanisms underlying these disorders and possible targets for interventions.

## Method

### Literature Search and Study Selection

For this systematic review, we followed the Preferred Reporting Items for Systematic Reviews and Meta-Analyses guidelines (Liberati et al., 2009). A research librarian conducted searches using CINAHL, Embase, PsycInfo, PubMED, Scopus, and the Cochrane Library. We selected a list of keywords based on a preliminary search of the literature and medical subject headings that included permutations of the following terms: *taste* OR *gustatory perception* OR gustat* AND *anorexia nervosa* OR *bulimia nervosa* OR *binge eating disorder* OR *eating disorder(s)*. Our inclusion criteria were as follows: published original research article in peer-reviewed journals in English, used human participants, published between database inception and July 2018, reported on patients meeting full criteria for an eating disorder, and assessed taste in people with an eating disorder. We excluded studies on cancer-related anorexia. We also performed a review of reference lists of included publications. Two authors independently reviewed titles, abstracts, and full-text articles for relevance, and a third author resolved any discrepancies.

### Data Extraction

For each article, an author (AMC, AR, and/or PVJ) independently extracted data using a structured tool that contained sections for participant and study characteristics (Supplemental Tables S1 and S2) and study results (Supplemental Table S3). Because of the large number of studies screened by title and abstract (*n* = 1490), two authors (AMC and PVJ) screened a random subset to establish reliability of the selection criteria, discussing and resolving discrepancies.

## Results

### Study Selection

**Figure 1.**
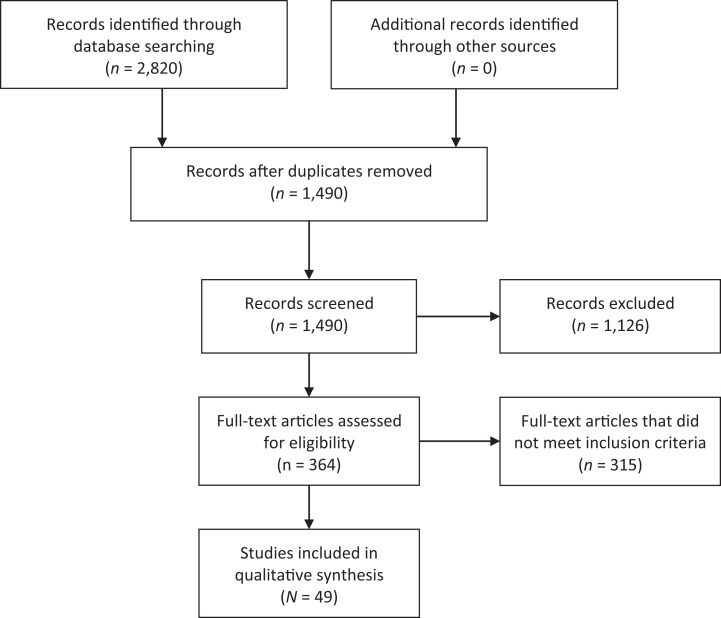
Preferred Reporting Items for Systematic Reviews and Meta-Analyses flow diagram of the literature screening process.

The initial search yielded 2,820 studies, 1,490 of which we assessed by title/abstract after removing duplicates. Of these, we assessed the full text of 364 articles and found that a total of 49 studies met the inclusion criteria (Figure 1). The diverse range of methodologies of the studies included in this systematic review and heterogeneity among studies limited our ability to complete a meta-analysis.

### Participant Characteristics

The number of participants per study ranged from *n* = 15 to *n* = 243 (Supplemental Table S1). The mean age in each study ranged from 14.8 to 42 years. Most studies (*n* = 43) included only female participants. Few studies (*n* = 7) explicitly stated the race/ethnicity of the sample. Approximately half of the studies (*n* = 29) were conducted in the United States. Studies included comparisons of patients with AN (*n* = 15), BN (*n* = 9), or BED (*n* = 4) versus controls and also versus patients with different eating disorders (e.g., AN vs. BN; *n* = 20). Studies also included a recovery definition (*n* = 15), but only eight studies included only fully recovered (remitted) participants.

### Study Designs

The majority of studies used a cross-sectional design (*n* = 42; Supplemental Table S2). Included articles consisted of psychophysical studies (*n* = 27) and studies using neuroimaging techniques such as electroencephalography (EEG) and functional magnetic resonance imaging (fMRI; *n* = 17). Tastes measured included sweet (*n* = 34), bitter (*n* = 13), salty (*n* = 10), and umami (*n* = 3), and 33 studies measured responses to other stimuli (i.e., fat, sour). Only 7 studies solely tested perception (sensitivity or recognition thresholds), while 11 focused only on taste preferences. In 14 studies, participants were required to fast, while 17 studies examined patients when they were satiated. Few studies controlled for confounding variables such as duration of illness or age of onset of eating disorder.

### Study Characteristics and Results

#### Self-report

Self-report studies primarily examined hedonics (*n* = 5), and each had distinct cohorts, sample sizes, aims, and methodologies. The largest study contained *n* = 243 participants (Koritar, Philippi, & Alvarenga, 2017); however, only 27 participants had BN, with the remainder divided between individuals without an eating disorder (*n* = 162) and those at risk for an eating disorder (*n* = 54). BN was the focus in two of these studies (Koritar et al., 2017; Mitchell et al., 1999), AN in two more (Monje Moreno, Alvarez Amor, Ruiz-Prieto, Bolanos-Rios, & Jauregui-Lobera, 2014; Steinglass, Foerde, Kostro, Shohamy, & Walsh, 2015), while the remaining study compared patients with AN or BN to controls (Brand-Gothelf et al., 2016).

One study utilized the Eating Hedonics Questionnaire to compare taste hedonics between participants with BED and BN (Mitchell et al., 1999). Participants with BED reported greater enjoyment of the smell, taste, and texture of food while binge eating compared to those with BN. Another study compared gustation between participants with AN and BN using the Sensory Responsiveness Questionnaire (SRQ), a self-report instrument (Brand-Gothelf et al., 2016). Most of these studies did not specifically use tastant solutions to assess taste; yet, investigators examining gustatory measures on the SRQ found that patients with AN had elevated sensory responsiveness and scores than people with BN (Brand-Gothelf et al., 2016). Some studies compared other eating disorder groups to controls while using different self-reported measures, including the Thought Fusion Questionnaire (TSF-Q; *n* = 3; Koritar et al., 2017; Monje Moreno et al., 2014; Steinglass et al., 2015). For example, Monje Moreno, Alvarez Amor, Ruiz-Prieto, Bolanos-Rios, and Jauregui-Lobera (2014) used the TSF-Q to compare reactivity between patients with AN and controls after exposure to and tasting of foods with different concentrations of sweetness and textures of fats. Patients with AN had significantly higher scores on the TSF-Q in postsweetness tasting than healthy controls, suggesting that patients with AN experienced greater cognitive distortion. Only two of these studies included a definition of recovery for those undergoing inpatient treatment (Steinglass et al., 2015) or receiving cognitive behavioral therapy (Mitchell et al., 1999), but the patients in these two studies were not considered remitted.

#### Psychophysical studies

The studies we categorized as psychophysical were based on taste preference or detection thresholds, with two studies examining both (Blazer, Latzer, & Nagler, 2008; Franko, Wolfe, & Jimerson, 1994). Despite the lack of consistency among studies, we could discern general trends. Of the nine studies that examined taste preference, three compared preferences between individuals with BN and controls, finding that patients with BN had a greater preference for sweetness (Drewnowski, Bellisle, Aimez, & Remy, 1987; Klein, Schebendach, Brown, Smith, & Walsh, 2009; Rodin, Bartoshuk, Peterson, & Schank, 1990). However, Drewnowski, Bellisle, Aimez, and Remy (1987) found that participants with BN showed an optimum preference for sweet stimuli that were low in fat concentration. Through the sip-and-spit methodology, Rodin, Bartoshuk, Peterson, and Schank (1990) showed that intensity ratings of sucrose (sweet), citric acid (sour), sodium chloride (salty), and quinine hydrochloride (bitter) on the palate were lower in those with BN compared to controls, but hedonic ratings did not differ between the two groups in other areas of the tongue. The remaining preference studies (Dazzi, Nitto, Zambetti, Loriedo, & Ciofalo, 2013; Goldzak-Kunik, Friedman, Spitz, Sandler, & Leshem, 2012; Pierce, Halmi, & Sunday, 1989; Simon, Bellisle, Monneuse, Samuel-Lajeunesse, & Drewnowski, 1993; Sunday & Halmi, 1990; Sunday & Halmi, 1991; Szalay et al., 2010) compared the taste preferences of people with AN to those of controls and/or people with BN. Of these, three studies (Pierce et al., 1989; Simon et al., 1993; Sunday & Halmi, 1990) showed that patients with either AN or BN had a strong aversion to fat compared to controls. Results varied among studies that compared individuals with AN to controls (Goldzak-Kunik et al., 2012; Simon et al., 1993; Szalay et al., 2010). Simon, Bellisle, Monneuse, Samuel-Lajeunesse, and Drewnowski (1993) found that participants with AN disliked fat more than did controls, but there was no difference in taste preference for sweetness. In another study, Goldzak-Kunik, Friedman, Spitz, Sandler, and Leshem (2012) found no evidence for reduced sensitivity to taste (sucrose, sodium chloride, quinine hydrochloride, monosodium glutamate, and citric acid) in patients with AN compared to controls. On the other hand, Szalay et al. (2010) found that those with AN had lower pleasantness scores for all five basic tastes compared to controls. Dazzi, Nitto, Zambetti, Loriedo, and Ciofalo (2013) confirmed that individuals with AN had overall poorer gustatory function and reduced perception of bitterness compared to healthy controls. Consistent with these findings, Sunday and Halmi (1991) demonstrated that participants with AN showed lower hedonic ratings for sweetness than patients with BN and controls.

Similar to the taste-preference studies, the cohorts and aims of the taste-threshold studies varied (Arlt, Smutzer, & Chen, 2017; Birmingham, Wong-Crowe, Hlynsky, & Gao, 2005; Jirik-Babb & Katz, 1988; Lacey, Stanley, Crutchfield, & Crisp, 1977; Nakai, Kinoshita, & Koh, 1987; Nozoe et al., 1996; Schebendach et al., 2014). In two of the taste-threshold studies, researchers found that patients with AN or BN had significantly lower detection levels when administered higher concentrations of sweet, salt, bitter, and sour solutions compared to controls, suggesting that AN and BN may lead to decreased taste sensitivity (Jirik-Babb & Katz, 1988; Nakai et al., 1987).

##### Pre-/posttreatment

A few (*n* = 4) of the psychophysical studies focused on measuring taste preferences and/or thresholds of patients with eating disorders before and after treatment. The majority of the pre-/posttreatment studies (*n* = 3) included participants with AN and BN as well as controls (Bossert et al., 1991; Drewnowski, Halmi, Pierce, Gibbs, & Smith, 1987; Sunday & Halmi, 1990). In addition, in most of these studies (*n* = 3; Drewnowski, Halmi, et al., 1987; Drewnowski et al., 1992; Sunday & Halmi, 1990), participants fasted prior to testing. Findings from one study showed that participants with AN and BN showed an aversion to fat before and after weight restoration, suggesting that this may be a common trait of these disorders (Drewnowski, Halmi, et al., 1987).

##### Other studies

Although the majority of psychophysical studies had cross-sectional designs, we also examined one that was longitudinal, one that was quasi-experimental, and two that were experimental (Aschenbrenner, Scholze, Joraschky, & Hummel, 2008; Drewnowski, Krahn, Demitrack, Nairn, & Gosnell, 1995; Eiber, Berlin, de Brettes, Foulon, & Guelfi, 2002; Peterson et al., 2016). Of these, the longitudinal study by Aschenbrenner et al. (2008) was the most robust and noteworthy. In this study, researchers used taste strips of varying concentrations of sweet, salt, bitter, and sour to analyze gustatory function in patients with AN or BN prior to admission to a hospital and during discharge. Although the patients were not fully recovered, the results indicated that patients with AN had more improved gustatory function over the course of therapy compared to patients with BN and healthy controls. However, at discharge, AN subjects still displayed lower gustatory test scores than healthy controls.

In addition, in two of the cross-sectional studies, researchers found no differences in sucrose pleasantness and sweetness between persons with remitted AN (RAN) and control women (CW; Frank et al., 2012; Frank, Shott, Keffler, & Cornier, 2016). Also, taste sensitivity did not differ among people with AN or those with obesity compared to controls; however, participants with AN showed opposite brain-reward responses from those with obesity (Frank et al., 2012). In another study, investigators found that perception of sucrose sweetness was higher among people with AN versus those with RAN but did not differ significantly between CW and people with BN (Frank, Shott, & Mittal, 2013). Conversely, in other studies, researchers found no difference in pleasantness between CW and individuals with AN, RAN, BN, or obesity (Frank, Reynolds, Shott, & O’Reilly, 2011; Frank, Shott, & Mittal, 2013; Monteleone et al., 2017). However, in one study, researchers found that sucrose pleasantness was lower in the AN group compared to the control group (Frank, Shott, Hagman, & Yang, 2013). In another study, women with AN found the cream solution significantly less pleasant than did CW (Radeloff et al., 2014).

#### Neuroimaging

Of the 17 studies, we reviewed that used neuroimaging techniques, most (*n* = 15) used fMRI, while the remainder (*n* = 2) used EEG. The majority of studies included sweet stimuli (*n* = 15). Wagner et al. (2008) reported that, when responses to sucrose and water were combined, patients with RAN showed reduced response over time in the bilateral insula, striatum including the dorsal and middle caudate, dorsal and ventral putamen, and anterior cingulate cortex (ACC) compared to controls. Consistent with these findings, in a later study, Wagner et al. (2015) found that patients with RAN demonstrated decreased sensitization to sucrose in the right medial frontal gyrus compared to controls, while those with remitted BN (RBN) demonstrated increased sensitization. In another study, the response to sucrose and sucralose was significantly less in individuals with RAN relative to CW in the right anterior and middle insula (Oberndorfer et al., 2013). Among adolescents with AN, orbitofrontal cortex volume was negatively correlated with sweet-taste pleasantness (Frank, Shott, Hagman, & Yang, 2013). Women recovered from BN had greater blood-oxygen-level-dependent signal responses to sucrose in the right anterior insula relative to CW, while those with RAN had a diminished response in the right anterior insula compared to CW (Oberndorfer et al., 2013). In response to sucralose, those with RAN had a decrease in right anterior insula activation compared to CW, while those with RBN and CW had responses that were similar to one another. In within-group analyses, sucrose elicited a greater response in the right caudate for women with RBN and a lower response in those with RAN compared to sucralose. However, another study demonstrated that CW had significantly higher activation compared with patients with RBN in the right ACC and left cuneus when glucose was compared to artificial saliva (Frank et al., 2006). Frank, Shott, Hagman, & Mittal (2013) analyzed gyrus rectus volume and found it to be positively correlated with sucrose pleasantness across the control and eating-disorder groups (i.e., AN, BN, RAN).

In five of the neuroimaging studies, researchers used a classical conditioning task where participants received 1 M of sucrose, no solution, or artificial saliva (Frank, Collier, Shott, & O’Reilly, 2016; Frank et al., 2012; Frank et al., 2011; Frank, Shott, Keffler, et al., 2016; Frank, Shott, Riederer, & Pryor, 2016). The results showed reduced neural response in the ventral putamen, insula, and orbitofrontal cortex in participants with BN compared to CW for both unexpected receipt and omission of taste reward (Frank et al., 2011). In contrast, participants with AN had increased brain activation in the orbitofrontal cortex when receiving unexpected reward and, compared to controls, had significantly stronger relationships with a computational temporal difference model that tests how brain response resembles dopamine neuronal function in the right insula, left thalamus, and left and right dorsolateral prefrontal cortex (Frank et al., 2012). Frank, Shott, Keffler, & Cornier (2016) reported greater posterior insula response in women with RAN than in controls to the unexpected stimulus omission but not for unexpected receipt. Classification accuracy in the insula for sucrose or artificial saliva versus no solution did not differ among groups (AN, CW, BN, obesity, and RAN). For sucrose versus artificial saliva, healthy controls and individuals with RAN demonstrated higher classification accuracy in the insula compared to those with AN or obesity (Frank, Shott, Keffler, et al., 2016). In the same study, researchers examined the effective connectivity in taste-reward and appetite-regulating pathways (e.g., posterior insula to middle orbitofrontal cortex, inferior orbiotofrontal cortex to prefrontal cortex) and found that pleasantness was not significantly correlated with brain results in any group after correction for false discovery rate.

In participants with AN, drinking chocolate milk while hungry compared to drinking it when satiated induced significantly greater activation in the right amygdala and the left medial temporal gyrus than it did in healthy controls (Vocks, Herpertz, Rosenberger, Senf, & Gizewski, 2011). Drinking chocolate milk during satiety compared with drinking it during hunger showed a significant difference in response in the left insula in healthy controls, whereas in participants with AN, there was a significant difference in neuronal activity in the inferior temporal gyrus, covering the extrastriate body area (Vocks et al., 2011). In another study, Bohon and Stice (2012) found that women with BN had a positive correlation between negative affect and activity in the putamen, caudate, and pallidum during a period of anticipation for receiving a chocolate milkshake; however, there was no significant relationship in healthy controls. Further, psychophysical interaction analyses revealed that activation in the amygdala showed a stronger association with activation in the left putamen and left insula during the period of anticipation of milkshake receipt in women with BN than in healthy controls. Toth, Kondakor, et al. (2004) used EEG to examine differences in responses between participants with AN and controls to sweet milk chocolate and bitter tea stimuli. Compared to controls, the AN group had higher amounts of relative θ in all 12 electrodes (Fp1, Fp2, F3, F4, C3, C4, T3, T4, T5, T6, P3, and P4), irrespective of taste condition. In a different analysis using the same participants (Toth, Tury, et al., 2004), the decrease in ω complexity resulting from exposure to sweet taste following exposure to bitter taste was significant on the left side among controls but was not significant in the AN group. Radeloff et al. (2014) found that the difference in response to cream versus water in the left anterior ventral striatum was significantly greater in those with RBN compared to those with RAN or to CW. Finally, compared to controls, patients with AN or BN had a decreased response to bitter stimuli in the right amygdala (Monteleone et al., 2017). There were no differences between healthy controls and AN or BN groups for sweet stimuli.

## Discussion

The primary aim of this review was to explore taste perception in people with eating disorders. Researchers have investigated taste perception in different types of eating disorders through self-report, psychophysical, and neuroimaging techniques. To the best of our knowledge, this is the first systematic review of studies conducted to assess taste differences among patients with AN, BN, and BED and health controls. Despite extensive research in eating disorders, comprehensive studies examining taste perception and its association with altered food consumption remain scarce. Our investigation yielded mixed results due to the heterogeneity of samples and methodolodgies; therefore, we could draw no firm conclusions about whether eating disorders affect taste perception or how taste perception might affect eating disorders. Despite the heterogeneity of populations studied and different methodological approaches used, however, we did observe some general trends.

### Inconsistency in Methods

The studies varied significantly on the methods used to measure taste perception (sensitivity or detection thresholds and hedonics). Studies used sip-and-spit tests, taste strips, food consumption, whole-mouth swallowed solutions, and spatial or regional tests. Researchers use whole-mouth taste tests to measure an individual’s ability to detect, evaluate, and identify the concentration of different sweet, sour, salty, and bitter taste solutions. The whole-mouth procedure involves sipping a measured volume of a taste solution, keeping it in the mouth for an allotted time, and spitting it out (Fushan, Simons, Slack, & Drayna, 2010). It is the most widely used technique in taste-testing chemosensory procedures. By contrast, spatial or regional tests enable evaluation of particular areas of the mouth using a cotton swab soaked in a taste solution and positioned in different areas of the tongue (Berling, Knutsson, Rosenblad, & von Unge, 2011; Hummel, Erras, & Kobal, 1997; Snyder, Prescott, & Bartoshuk, 2006). There was also a lack of consistency as to whether participants fasted prior to testing, and in many instances, authors did not explicitly state whether participants fasted prior to testing. Fasting is particularly important for decreasing confounding variables that might affect the test results. The range of concentrations of the tastants was not standardized among studies, and there was no consistency of reporting between studies (e.g., mM, M, g/mL, %). The sample sizes of the studies also varied (i.e., 15−243 subjects).

### Neural Responses

Results from neuroimaging studies demonstrated differences in neural responses when tastes were administered in a predictable versus an unpredictable way. These findings suggest that regions of the brain involved with taste reward interact with cognitive control processes that drive eating behaviors and choices. Thus, it may be that, with repeated and expected administration of sweet tastes, patients with AN anticipate the task and decrease neural response in order to prevent higher reward stimulation (Frank, 2015). Conversely, with random administration of tastants, such anticipation is not possible, and the higher responsiveness to sweet tastes is evident in patients with AN. With BN, however, researchers reported a reduced responsiveness during random application but not during repeated and predictable administration where participants might have been anticipating a hedonic reward.

### Inconsistent Findings in Self-Report and Psychophysical Studies

The literature, as of now, is conflicting and there are no clear answers from self-report and psychophysical studies as to how taste perception and detection thresholds are impacted by or impact eating disorders, with some findings indicating that taste function is impaired in patients with eating disorders and others indicating it is not. Some investigators found decreased or altered taste sensitivity in AN and BN participants relative to healthy controls (Aschenbrenner et al., 2008; Drewnowski, Halmi, et al., 1987; Nakai et al., 1987), which might affect eating behavior.

Several factors could affect taste perception in individuals with eating disorders. For example, researchers have observed a decrease in fungiform papillae on the tongue in persons with AN, and in persons with BN, the consistent purging may change the oral microenvironment, affecting taste buds and saliva composition (Rodin et al., 1990). Other studies reported conflicting results, however, suggesting that taste alterations were a result of decreased body weight, malnutrition, metabolic problems, or fear of food-related stimuli (Dazzi et al., 2013; Di Costanzo et al., 1998; Goldzak-Kunik et al., 2012; Vocks et al., 2011), with some studies showing improvement of taste alterations after weight recovery (Aschenbrenner et al., 2008) or behavioral interventions (Nozoe et al., 1996). From our examination of self-report studies, we found that participants’ taste perceptions were relatively unchanged after weight restoration (Brand-Gothelf et al., 2016). This finding was consistent with our analysis of pre-/posttreatment studies, which examined the effects of weight restoration on taste perception and detection in AN and BN populations. These findings call into question the proposal that BMI plays a role in taste perception (Feeney, O’Brien, Scannell, Markey, & Gibney, 2011).

In addition, studies reported great variation in the perceptions of different tastes such as sweetness and fat. For example, results from this review indicated that individuals with BN have a greater preference for sweetness compared to control groups. These differences may be due to several factors such as variances in basic human biology (e.g., variation in the human *TAS1R2* or *TAS1R3* gene associated with sweet taste [Kim, Wooding, Riaz, Jorde, & Drayna, 2006]) or cognitive processing for a perceived stimuli (Webb, Bolhuis, Cicerale, Hayes, & Keast, 2015). Some results revealed shared characteristics across eating disorders (e.g., strong aversion toward fat; Hoogeveen et al., 2015). Such characteristics may be the result of social stigma against fatty foods or pathophysiology in eating-disorder populations.

### Cognition and Taste

Researchers reported differences in taste responses between swallowed and nonswallowed solutions in individuals with AN, which suggests that the anticipation of ingesting calories or anxiety around gaining weight, rather than a diminished ability to experience gratification, led to a change in taste perception (Eiber et al., 2002; Monje Moreno et al., 2014). This finding underscores the potential role of cognitive factors in taste perception in patients with AN. Future investigations should implement measures that assess how and when cognition influences taste perception and pathophysiology in AN. Individuals with BN reported decreased taste intensity compared to healthy controls (Jirik-Babb & Katz, 1988; Nakai et al., 1987). Further, they experienced decreased taste sensitivity when solutions were placed on the tongue using taste strips rather than being administered using the whole-mouth method (Aschenbrenner et al., 2008; Dazzi et al., 2013; Rodin et al., 1990). However, there were discrepancies in methods and findings across studies, and it is unclear whether the taste alterations were the result of BN or of another risk factor.

Alteration of the reward-processing mechanisms also plays an important role in eating disorders and contributes to the development and maintenance of symptoms (Berridge, 2009; Kaye, Fudge, & Paulus, 2009; Keating, Tilbrook, Rossell, Enticott, & Fitzgerald, 2012). Neuroimaging allows for quantitative evaluation of mechanisms associated with reward processing in eating disorders and may provide insights into potential similarities and/or differences between related conditions. A neuroimaging paradigm that researchers often use in studies on eating disorders comprises looking at brain-reward pathways while individuals taste a pleasant food (e.g., fat, sugar). In the neuroimaging studies we reviewed, findings suggest that individuals with AN have distinctive alterations in the insula, which is the section of the brain associated with taste identification (Frank, Collier, et al., 2016; Monteleone et al., 2017; Wagner et al., 2008). These findings also suggest that there may be alterations in processing information associated with self-awareness in individuals recovering from AN compared to controls. Toth, Tury, et al. (2004) found that persons with AN had different patterns of brain activation when given pleasant (i.e., sweet) or unpleasant (i.e., bitter) stimuli compared to controls, suggesting that they struggle to identify taste or respond to the hedonic appeal associated with food. In addition, since the insula contributes to emotional regulation, it is possible that, in individuals with AN, food generates an aversive rather than a rewarding response. This finding might provide further insight into why individuals with AN avoid typically “pleasurable” foods.

### Research Gaps

None of the studies we reviewed included the possible effects of individual genetic variation on the perception of the taste stimulus (e.g., bitter and *TAS2R38*) or tested whether individuals with eating disorders have reduced numbers of taste papillae, especially those with BN or BED, which could contribute to altered taste perception. Most studies used only one measure of taste function to quantify each stimulus. Studies also did not explicitly consider other confounding factors, such as nutrition, that could play a role in gustatory function in eating-disorder populations. In addition, researchers overlooked some potential factors in the psychophysical studies. For example, there were a limited number of studies among remitted patients. By analyzing the effects of recovery from eating disorders on taste function, we might be able to better characterize such disorders and identify risk of relapse. Another variable that the studies commonly overlooked was weight: Almost all studies lacked control for differences in weight and starvation and their possible influence on measures of taste perception. In addition, researchers frequently did not adjust for duration of illness. This factor is particularly important since taste may be affected by whether someone’s eating disorder(s) has recently started or persisted for many years. Future studies should include remitted patients, and psychophysical studies in particular should control for weight and duration of illness.

## Limitations

Although we conducted a comprehensive systematic review of differences in taste perception, preference, and response across multiple eating disorders (AN, BN, and BED), we were unable to conduct a meta-analysis. Although there were several studies in each group (i.e., self-report, psychophysical, neuroimaging), the heterogeneity of the studies made it difficult to draw strong conclusions. For example, the self-report studies utilized different questionnaires and taste measures and were conducted among different eating-disorder populations. Standardizing the methodology for taste assessment among specific cohorts of eating-disorder patients would likely provide more consistent and comparable findings.

## Conclusions

In this article, we reviewed studies that addressed taste perception in eating-disorder populations. The need for a gold standard methodology to measure taste perception in eating disorder is clear. Such standardization would allow future reviews and meta-analyses to draw stronger, potentially clinically relevant, conclusions. More research is needed, as well, on the biological factors that might influence taste, such as genetics. Improved understanding of differences in taste perception and preferences in eating-disorder populations and in the mechanisms that underlie these differences could have implications for treatment and prevention of these disorders.

## Supplemental Material

Supplemental Material, Chao_19040064_toSage_SuppTbls - A Systematic Review of Taste Differences Among People With Eating Disorders Click here for additional data file. Supplemental Material, Chao_19040064_toSage_SuppTbls for A Systematic Review of Taste Differences Among People With Eating Disorders by Ariana M. Chao, Abhrarup Roy, Alexis T. Franks and Paule V. Joseph in Biological Research For Nursing
