# Supplementary material for: A Systematic Review of Taste Differences Among People With Eating Disorders
Source: Biol Res Nurs. 2019 Sep 4;22(1):82–91. doi: 10.1177/1099800419872824 (PMC6912928; doi:10.1177/1099800419872824)
Supplement: Supplemental Material, Chao_19040064_toSage_SuppTbls - A Systematic Review of Taste Differences Among People With Eating Disorders [file Chao_19040064_toSage_SuppTbls.pdf]

Table S1. Participant characteristics of the reviewed literature

Citation	Total Sample	# Anorexia	# Bulimia	#BED	#Control	Age	BMI	Race/ethnicity	%Female	Eating Disorder Defined	Control Condition Definition	Recovery Definition	Duration of Recovery	Duration of Illness in Years (mean±SD)	Age of Onset
								Psychophysical Studies							
Arlt, Smutzer, and Chen (2017)	60	N/A	N/A	Normal Weight Binge Eating Disorder: 15 Overweight Binge Eating Disorder: 15	30 (15 normal weight controls, 15 overweight controls)	Normal Weight Control: 21.38±3.12 Normal Weight Binge Eating Disorder: 21.69±3.18 Overweight Control: 35.27±11.46 Overweight Binge Eating Disorder: 35.27±11.46	Normal Weight Control: 21.62±1.56 Normal Weight Binge Eating Disorder: 22.29±1.88 Overweight control: 30.74± 3.26 Overweight Binge Eating Disorder: 33.06±3.39	Normal Weight Control: 12 White, 2 Asian, 1 Hispanic Normal Weight Binge Eating Disorder: 12 White, Black/African American: 1, Asian: 2, Overweight Controls: 5 White, 9 Black/African American, and 1 Hispanic, Overweight Binge Eating Disorder: 5 White, 8 Black/African American, 1 Hispanic, 1 Other	100%	DSM-IV	Recruited through paper and online advertisements. Normal and overweight controls	N/A	N/A	N/A	N/A
Aschenbrenner, Scholze, Joraschky, and Hummel (2008)	63	16	24	N/A	23	24.5±4.0	14.94±2.05	N/A	100%	DSM-IV and DIA-X/M-CIDI	Healthy females who didn't score positive in eating disorder questionnaire	N/A	N/A	Anorexia Nervosa: 6.9 Bulimia Nervosa: 4.8	N/A

Birmingham, Wong-Crowe, Hlynsky, and Gao (2005)	16	N/A	N/A	N/A	N/A	31±13	N/A	N/A	100%	100%	N/A	N/A	N/A	N/A	N/A
Blazer, Latzer, and Nagler (2008)	52	N/A	26	N/A	26	24±7	Bulimics: 22.3, No Standard Deviation Controls: 21.9, No Standard Deviation	N/A	100%	DSM-IV	Healthy Volunteers	N/A	N/A	N/A	N/A
Bossert et al. (1991)	33	7	17	N/A	9	Anorexia: 21.9±2.9 Bulimia Nervosa: 21.7±2.9 Control: 22.3±1.2	N/A	N/A	100%	DSM-III	Healthy women that were examined at their maximum amount of weight loss after a four-week period	Behavior therapy	8 Weeks	Anorexia: 4.4±3.4 Bulimia: 5.5±2.9	N/A
Dalton and Finlayson (2014)	N/A	N/A	N/A	Not Specified	N/A	N/A	Lean and obese participants	N/A	100%	DSM-V	N/A	N/A	N/A	N/A	N/A

Dazzi, Nitto, Zambetti, Loriedo, and Ciofalo (2013)	56	18	19	N/A	19	Eating Disorder Group: 26.7±2.0 Control: 27.6 (no Standard Deviation)	Anorexia Nervosa: 15.74 Bulimia Nervosa: 22.07	N/A	Eating Disorder Group: 100% female<br><br>Control: 14/19 female (74%)	DSM-IV	Medical students with no taste & smell disorders, ear, nose and throat diseases, or eating/psychiatric disorders	N/A	N/A	N/A	N/A
Drewnowski, Bellisle, Aimez, and Remy (1987)	32	N/A	16	N/A	16	Bulimia Nervosa: 26.7±2.0 Control: 28.9±1.8	Bulimia Nervosa: 20.7±0.3 Control: 20.9±0.6	N/A	100%	DSM-III	No Eating Disorders; recruited among students from University of Paris	N/A	N/A	N/A	N/A
Drewnowski, Halmi, Pierce, Gibbs, and Smith (1987)	48	12	20 (13 Anorectic Bulimic and 7 Bulimic)	N/A	16	Anorexic Nervosa-Restrictor: 16.3±2.2 Anorectic-Bulimic: 19.5±4.2 Bulimic: 19.4±2.5 Control: 19.1±0.8	Anorexia Nervosa-Restrictor: 14.8±1.6 Anorectic-Bulimic: 16.2±2.3 Bulimic: 21.3±2.0 Control: 21.1±1.6	N/A	100%	DSM-III	Normal-weight females and free of Eating disorders; recruited from university campus	Liquid diet given to ensure patient gained 2 lb/week until patient reached target weight of 50th percentile	Eight Weeks	N/A	N/A
Drewnowski, Krahn, Demitrack, Nairn, and Gosnell (1992)	26	N/A	N/A	14	12	Binge Eating Disorder: 26.3±2.0 Control: 31.9±2.0	Binge Eating Disorder: 30.9±2.2 Control: 22.7±0.6	N/A	100%	DSM-III	Recruited through advertisements and had no history of eating disorders	N/A	N/A	N/A	N/A

Drewnowski, Krahn, Demitrack, Nairn, and Gosnell (1995)	41	N/A	N/A	20	21	Obese: 28.1±7.0<br>Lean: 27.4±8.0<br>Binge Eaters: 25.4±7.4<br>Non-Bingers: 29.8±7.3	Binge Eaters: 29.2±7.9<br>Non-Bingers: 26.4±6.3<br>Obese: 35.1±6.0<br>Lean: 23.0±2.0	N/A	100%	DSMIII & IV	41 participants total. Of these participants, there was overlap between non-bingers (21) and Lean individuals (25)	N/A	N/A	N/A	N/A
Eiber, Berlin, de Brettes, Foulon, and Guelfi (2002)	60	Restrictive Anorectics: 20<br>Anorectic-bulimics: 20	Bulimics: 20	N/A	N/A	Restrictive-Anorectics: 23.3±4.8<br>Bulimics: 26.4±5.5<br>Anorectic-Bulimics: 26.4±5.5	Restrictive-Anorexics: 15.7±1.6<br>Bulimics: 22.7±2.7<br>Anorectic-Bulimics: 16.3±1.3	N/A	100%	DSM-IV	N/A	N/A	N/A	N/A	N/A
Franko, Wolfe, and Jimerson (1994)	40	N/A	20	N/A	20	Bulimia Nervosa with a history of Anorexia: 25±4<br>Bulimia Nervosa with no history of Anorexia: 24±5<br>Healthy Controls: 24±4	N/A	N/A	100%	DSM III-R	Controls selected based on absence of personal history or family history of eating disorders, other major psychiatric disorders, and obesity	N/A	N/A	Bulimia Nervosa with history of Anorexia: 10±6<br>Bulimia Nervosa with no history of anorexia: 6±5<br>Overall average of BN: 7±5	N/A

Goldzak-Kunik, Friedman, Spitz, Sandler, and Leshem (2012)	30	15	N/A	N/A	15	Control: 15.0±0.48 Anorexic: 15.8±0.34	Control: 19.4±0.58 Anorexic: 17.2±0.50	N/A	93%	Diagnosed by inpatient medical team	Healthy group consisting of 14 Females and 1 Male below the 65th BMI percentile	N/A	N/A	N/A	N/A
Jirik-Babb and Katz (1988)	21	9	5	N/A	7	N/A	N/A	N/A	100%	DSM-III	Age-matched, non-hospitalized, normal-weight, non-eating disordered women	N/A	N/A	N/A	N/A
Klein, Schebendach, Gershkovich, Smith, and Walsh (2010)	24	N/A	13	N/A	11	Bulimia Nervosa: 24.45±1.55 Control: 26.7±1.4	Bulimia Nervosa: 22.46±0.83 Control: 21.01±0.66	N/A	100%	Met DSM-IV criteria for Bulimia Nervosa	Healthy women with no history of an eating disorder	N/A	N/A	Bulimia Nervosa: 9.7±2.3	N/A
Lacey, Stanley, Crutchfield, and Crisp (1977)	12	6	N/A	N/A	6	Anorexia Nervosa: 245.4 months±60.9 Control: 253.2±83.2	N/A	N/A	100%	N/A	Healthy female nurses	N/A	N/A	N/A	N/A

Nakai, Kinoshita, and Koh (1987)	36	23	13	N/A	N/A	Anorexia Nervosa: 19.3±4.0 Bulimia Nervosa: 21.5±4.0	IBW: Anorexia Nervosa - 70.9±4.8 Bulimia Nervosa - 88±11.2	Japanese	100%	Research Group for Intractable Disease from the Ministry of the Health and Welfare of Japan	N/A	N/A	N/A	N/A	N/A
Nozoe et al. (1996)	15	9	N/A	N/A	6	Anorexia Nervosa: 19.3±3.8	N/A	Japanese	100%	Research Group for Intractable Disease from the Ministry of the Health and Welfare of Japan	Healthy females who were Age and sex matched	N/A	N/A	21.4±11.1	N/A

Peterson et al. (2016)	30	N/A	N/A	N/A	15	15.27±1.91	17.69±2.28	White, Non-Hispanic	100%	Inpatient Medical Care team; No DSM mentioned	Healthy female controls with no eating disorder. Must have low BMI percentile for age and height and stable body weight. Controls were matched to the eating disorder group on age (±6 months ED girls' age), age at menarche (age at menarche ± 12 months of eating disorder girl's age at menarche), race, weight (BMI percentile within 20% of eating Disorder match), and socioeconomic status (SES)	N/A	N/A	N/A	N/A

Pierce, Halmi, and Sunday (1989)	103	33 Anorectic Restrictors, 32 Anorectic Bulimics	24	N/A	24	N/A	N/A	N/A	N/A	N/A	N/A	N/A	N/A	N/A	N/A
Rodin, Bartoshuk, Peterson, and Schank (1990)	32	N/A	16	N/A	16	Control: 22.6 Bulimic: 23.4	N/A	N/A	100%	DSM-III-R	Drawn from a random sample of age-matched volunteers. Did not differ from bulimics in race, ethnicity or SES. Subjects paid $10.	N/A	N/A	At least three years	N/A
Schebendach et al. (2014)	50	25	N/A	N/A	25	Anorexia Nervosa: 27.2±7.8 Control: 23.3±3.5	Control: 21.1±1.6 Anorexia Nervosa: 17.3±2.0	Anorexia Nervosa = 2 Hispanics and 23 Caucasians, Normal controls were all Caucasians	100%	DSM-V	Healthy	N/A	N/A	N/A	N/A

Simon, Bellisle, Monneuse, Samuel-Lajeunesse, and Drewnowski (1993)	26	12	N/A	N/A	14	26.8	Anorectic: 14<br>Control: 20.7	N/A	100%	DSM-III-R	Medical students and nursing staff at the Hospital Ste Anne, Paris	Undergoing treatment in in-patient setting but patients were not recovered	N/A	8.9 years	N/A
S. R. Sunday and Halmi (1990)	151	Anorexic Restrictor: 48<br>Anorexic-Bulimic: 36	Normal Weight Bulimic: 42	N/A	Normal Weight Control: 26	Anorexia Nervosa-Restrictor: 18.94±0.85<br>Anorexia Nervosa-Bulimic: 21.31±0.80<br>Normal Weight Bulimic: 21.51±0.89<br>Normal Weight Control: 19.27±0.24	**Pre-BMI -** Anorexia Nervosa-Restrictor: 14.68±0.27<br>Anorexia Nervosa-Bulimic: 16.30±0.36<br>Normal Weight Bulimic: 20.75±0.31<br>Normal Weight Control: 20.91±0.33	N/A	100%	DSM-IIIR	Normal weight	N/A	N/A	N/A	N/A
Suzanne R. Sunday and Halmi (1991)	82	24 only Anorexic<br>26 Anorexic AND Bulimic	17	N/A	15	N/A	N/A	N/A	N/A	DSM-IIIR	Controls had no history an eating disorder	Received treatment in in-patient setting, tested during first week of admission and prior to discharge	Six Weeks	N/A	N/A

Szalay et al. (2010)	22	Anorexia Nervosa: 11 (10 women, 1 man)	N/A	N/A	Healthy Control: 11 (9 women, 2 men)	Anorexia Nervosa: 23, No SD Control: 24, No SD	Anorexia Nervosa: 16.7±1.6 Control: 22.8±1.9	N/A	83%	DSM-IV	Healthy females Age and sex matched	All subjects returned to normal body weight and all bulimics were free of bingeing and purging for at least four weeks	N/A	N/A	N/A
							Self-Report								
Brand-Gothelf et al. (2016)	Study 1 - 67 Study 2 - 20	40	20	N/A	27	Study 1 - Anorexia Nervosa-Restrictor: 17.8±5.0 Bulimia Nervosa-Restrictor: 22±4.7 Control = 18.5±4.7 Study 2 - Anorexia Nervosa-Restrictor: 15.7±1.6	Study 1 - Anorexia Nervosa-Restrictor: 15.7±1.6 Bulimia Nervosa: 20.8±2.8 Control: 20.2±2.8; Study 2 - Time of admission: 15.3± 1.6, Time of discharge: 19.6±1.7	N/A	100%	Eating Disorders Family History Interview (EDFHI) and DSM-IV	Between Ages 15-25, matched for age, years of schooling, ethnic origin, and catchment area	Group of Anorexics (n = 20) assessed on admission and discharge after weight restoration	2 weeks	Anorexics: 2.2±1.1 Bulimics: 2.6±3.2	N/A

Koritar, Philippi, and Alvarenga (2017).	243	N/A	27	N/A	College students without eating disorder risk: 162<br><br>College students with eating disorder risk: 54	Bulimia Nervosa: 26.2±6.9<br>College students without eating disorder risk: 21.9±4.3<br>College students with eating disorder risk: 21.8± 2.6	Bulimia Nervosa: 27.5±6.6<br>College students without eating disorder risk: 22.4±3.8<br>College students with eating disorder risk: 23.7±3.5	N/A	100%	DSM-IV	Recruited 216 healthy samples who took an eating disorder survey (EAT-26)	N/A	N/A	N/A	N/A
Mitchell et al. (1999)	78	N/A	29	49	N/A	Bulimia Nervosa: 26<br>Binge Eating Disorder: 42	Bulimia Nervosa: 23.5<br>Binge Eating Disorder: 34.3	Bulimia Nervosa: 93.1% Caucasian<br>Binge Eating Disorder: 98% Caucasian	100%	DSM-IV patient version	N/A	Receiving Cognitive Behavioral Therapy for Binge Eating Disorder but not recovered	N/A	N/A	N/A
Monje Moreno, Alvarez Amor, Ruiz-Prieto, Bolanos-Rios, and Jauregui-Lobera (2014)	38	23	N/A	N/A	15	21.34±4.96	N/A	N/A	100%	DSM-IV	Students recruited from the Pablo de Olavide University, Seville. Students did not have any psychiatric history	N/A	N/A	N/A	N/A

Steinglass, Foerde, Kostro, Shohamy, and Walsh (2015)	42	21 (Data from one individual with AN were excluded from analyses due to failure to understand the task instructions.)	N/A	N/A	20	Control: 26.3±5.8 Anorectic: 29.4±11.2	N/A	N/A	95% females in control group; 95.2% females in anorexic group	DSM-V	Healthy controls had no current or past psychiatric illness, including any history of an eating disorder, and had a BMI greater than or equal to 18.5	Undergoing treatment in in-patient setting but not recovered	N/A	N/A	N/A
Imaging Studies															
Bohon and Stice (2012)	26	N/A	13	N/A	13	20.3±1.87	23.6±2.6	4% Hispanic, 80% Caucasian, 12% Asian, 4% African American	100%	Completed Eating Diagnostic interview; DSM-V	Assessed with Eating Disorder Diagnostic Interview	N/A	N/A	N/A	N/A
Frank et al. (2006)	16	0	10 (all recovered)	0	6	Bulimia Nervosa-Restrictor: 27.5±7; Control: 22±2	Bulimia Nervosa-Restrictor: 22±3; Control: 21±2	N/A	100	N/A	N/A	Not reported	>/=1 year; 45±60 months (range of 12-180 months)	N/A	N/A
Frank, Reynolds, Shott, and O'Reilly (2011)	43	0	20	0	23	BN: 25.2±5.3; Control: 27.2±6.4	Bulimia Nervosa: 22.6+5.7; Control: 21.5+1.2	N/A	100	DSM-IV	Healthy	N/A	N/A	74.2+63.7 Months	N/A
Frank et al. (2012)	63	21	0	0	19 Obese; 23 Control	Control: 24.8±5.6; Anorexia Nervosa: 22.5±5.8; Obese: 27.1±6.7	Control: 21.5±1.4; Anorexia Nervosa: 16.1±1.1; Obese: 34.7±4.9	N/A	100	DSM-IV	Obese: BMI≥30 kg/m2, no psychiatric disorder, not taking medication; Control: Healthy	N/A	N/A	N/A	N/A

Frank, Shott, Hagman, and Yang (2013)	41	Anorexia Nervosa (Adolescents): 19; Anorexia Nervosa (adults): 19	0	0	Control: 22 Control (Adults): 24	Anorexia Nervosa (Adolescents): 15.4±1.4; Control(Adolescents): 14.8±1.8; Anorexia Nervosa Adults: 23.1±5.8; Control (Adults): 27.4±6.3	Anorexia Nervosa-(Adolescents): 16.2±1.1; Control (Adolescents): 21.3±1.9	N/A	100%	DSM-IV	Healthy	N/A	N/A	N/A	N/A
Frank, Shott, and Mittal (2013)	86	Anorexia Nervosa: 19; Anorexia Nervosa-Restrictor: 24	19	0	24	Control: 27.4±6.4; Anorexia Nervosa: 23.1+5.8; Anorexia Nervosa-Restrictor: 30.3+8.1; Bulimia Nervosa: 25.2+5.3	Control: 21.6+1.3; Anorexia Nervosa: 16.0+1.1; Anorexia Nervosa-Restrictor: 20.8+2.4; Bulimia Nervosa: 22.6+5.7	N/A	100	DSM-IV	No history of head trauma, neurological disease, major medical illness, psychotic or substance use disorder	≥1-year normal weight for height, menstrual cycles, exercise, and food intake	N/A	N/A	N/A
Frank, Collier, Shott, and O'Reilly (2016)	48	24 (recovered)	0	0	24	Anorexia Nervosa-Restrictor: 30.3±8.1; Control: 27.4±6.3	Anorexia Nervosa-Restrictor: 20.8±2.4; Control: 21.6±1.3	N/A	100	DSM-IV	N/A	Healthy body weight (18.5-24.9), menstrual cycle, exercise regime, and food intake for 1 year	7.9±6.0	5.9±5.2	16.6±2.4

Frank, Shott, Riederer, and Pryor (2016)	77	26	25	0	26	Control: 24.4±3.5; Anorexia Nervosa: 23.2±5.3; Bulimia Nervosa: 24.6±4.2	Control: 21.6±1.2; Anorexia Nervosa: 16.2±1.1; Bulimia Nervosa: 23.6±5.9	N/A	100	DSM- IV	Without history of head trauma, neurological disease, major medical illness, psychosis or substance-use disorders	N/A	N/A	Anorexia Nervosa: 6.6±5.7; Bulimia Nervosa: 7.1±4.5	N/A
Frank, Shott, Keffler, and Cornier (2016)	106	21; 19 (Recovered)	20	0	27 Healthy Control; 19 Obese Control	Control: 26.2±7.0; Anorexia Nervosa: 23.1±6.1; Anorexia Nervosa-Restrictor: 27.0±5.3; Bulimia Nervosa: 25.2±5.3; Obese: 28.2±8.1	Control: 21.5±1.4; Anorexia Nervosa: 16.0±1.1; Bulimia Nervosa: 22.6±5.7; Obese: 34.7±4.6; Anorexia Nervosa-Restrictor: 20.2±1.1	N/A	100%	DSM-IV	No history of psychiatric or major medical illness, were not taking medication, and were within normal BMI range life long	Normal weight, regular menses, normal exercise patterns for ≥1 year	N/A	N/A	N/A
Monteleone et al. (2017)	60	20	20	0	20	Control: 27.1±4.7; AN=25.5±7.8; Bulimia Nervosa: 27.7±8.0	Control: 21.0±1.5; AN: 17.3±1.0; Bulimia Nervosa: 21.8±2.6	N/A	100	DSM-V	No history of eating disorders; age ≥18 years; no psychopharmacological treatment during the preceding 6 weeks; no history of neurological or medical diseases and drug abuse dependence; no history of head trauma with loss of consciousness; no concomitant	N/A	N/A	Anorexia Nervosa: 7.6±6.7; Bulimia Nervosa: 8.8±6.1	Anorexia Nervosa: 16.5±2.8; Bulimia Nervosa: 18.3±4.0

											comborbid axis I psychiatric disorders				
Oberndorfer et al. (2013)	42	14 (recovered)	14 (recovered)	0	14	Control: 27.4±5.5; Anorexia Nervosa-Restrictor: 27.3±1.4; Bulimia Nervosa-Restrictor: 26.6±5.7	Control: 22.6±1.5; Anorexia Nervosa-Restrictor: 21.5±2.8; Bulimia Nervosa-Restrictor: 22.9±2.1	N/A	100	DSM-IV	No history of an eating disorder or other psychiatric disorder; no history of serious medical or neurological illness; no first-degree relatives with an eating disorder; within normal weight range since menarche	No restrictive eating or other eating disorder related behaviors in the preceding year; stable weight between 90% and 120% anorexic body weight for at least 1 year; regular menstrual cycles for 1 year; normal plasma b-hydroxybutyric acid, glucose, and insulin	Anorexia Nervosa-Restrictor =5.0±1.6; Bulimia Nervosa-Restrictor: 22.7±1.3	Anorexia-Nervosa-Restrictor: 8.2±1.7; Bulimia Nervosa-Restrictor: 8.0±5.9	N/A
Radeloff et al. (2014)	47	15 (all recovered)	14 (recovered)	0	18	Control: 24.7±3.1; Anorexia Nervosa-Restrictor: 25.2±4.0; Bulimia Nervosa-Restrictor: 24.5±2.4	Control: 21.5±1.8; Anorexia Nervosa-Restrictor: 21.0±2.4; Bulimia Nervosa-Restrictor: 20.6±2.1	N/A	100	DSM-IV	No history of an eating disorder or any psychiatric, serious medical or neurological illness; no first-degree relatives with an eating disorder; normal menstrual cycles; normal	Normal BMI; regular menstrual cycles; not engaged in pathological eating behavior	>/=1 year; Anorexia Nervosa-Restrictor: 72.4±31.3 months); Bulimia Nervosa Restrictor: 41.1±28.0 months	N/A	Includes

											weight range since menarche; no medication				
Toth, Kondakor, et al. (2004)	19	Anorexia Nervosa: 9	N/A	N/A	Control: 10	Anorexia Nervosa: No mean, Range was 18-30, No Standard Deviation Healthy Control: Range was 18-27, No Standard Deviation	N/A, mentioned being normal weight	N/A	100%	DSM-IV	Healthy	N/A	N/A	N/A	N/A
Toth, Tury, et al. (2004)	18	9 (7 women)	N/A	N/A	9 (4 women)	Anorexia Nervosa: Range was 18-30, No Standard Deviation Healthy Control: Range was 18-27, No Standard Deviation	N/A	N/A	61.10%	N/A	Healthy college students	N/A	N/A	N/A	N/A

Vocks, Herpertz, Rosenberger, Senf, and Gizewski (2011)	24	12	0	0	12	Anorexia Nervosa: 27.4±10.6; Control: 25.3±3.6	Anorexia Nervosa: 14.1±1.8; Control: 21.4±1.6	N/A	100	DSM-IV-TR	Controls have no current or past mental or physical disorder	N/A	N/A	N/A	N/A
Wagner et al. (2008)	32	16 (recovered)	0	0	16	Control: 26.8±6.3; Anorexia Nervosa-Restrictor: 26.4±6.2	Control: 22.9±2.2; Anorexia Nervosa-Restrictor: 20.7±2.2	N/A	100	DSM-IV	No history of an eating disorder or any psychiatric, serious medical, or neurological illness; no first-degree relative with an eating disorder; normal weight since menarche; normal menstrual cycles	Weight >90% anorexic body weight; regular menstrual cycles; have not binged, purged, or engaged in significant restrictive eating patterns for at least 1 year	45.7±30.2 months	N/A	Includes

Wagner et al. (2015)	42	14 (all recovered)	15 (recovered)	0	13	Control: 26.3±5.9; Anorexia Nervosa-Restrictor: 26.4±5.4; Bulimia Nervosa-Restrictor: 27.4±6.8	Control: 22.4±1.5; Anorexia Nervosa-Restrictor: 20.9±2.8; Bulimia Nervosa-Restrictor: 22.5±2.0	N/A	100	N/A	No history of an eating disorder or any psychiatric or neurological illness; no first-degree relatives with an eating disorder; normal weight since menarche	No restrictive eating or other eating disorder related behaviors in the preceding year; stable weight between 90% and 120% ABW for at least 1 year; regular menstrual cycles for 1 year; normal plasma b-hydroxybutyric acid, glucose, and insulin	Anorexia Nervosa-Restrictor: 58.4±69.4 months; Bulimia Nervosa-Restrictor: 44.3±47.2 months	N/A	Includes

Table S2. Study characteristics from the reviewed literature

Citation	Study Design	Study Methodology	Study's Primary Purpose	Taste					Taste Test Conducted	Fasted	Satiated	Whole mouth vs localized
				**Sweet**	**Salty**	**Bitter**	**Umami**	**Other stimulus**				
						Psychophysical Studies						
Arlt et al. (2017)	Cross-sectional	Psychophysical	To show interaction between weight status and Binge Eating Disorder. Additionally, to show differences in taste perception from non-eating disorder matched groups.	Sucrose: 2.5% and 5%	N/A	6-n-propylthiouracil (PROP): 0.11%	N/A	N/A	NIH Toolbox Gustatory Assessment (tests for whole mouth and regional taste sensitivity) & Edible Taste Strips	N/A	N/A	Regional and whole mouth
Aschenbrenner et al. (2008)	Longitudinal Prospective	Psychophysical	To employ detailed olfactory and gustatory testing in female subjects of three homogenous groups – anorexia nervosa, bulimia nervosa and healthy controls – and to look at the effects of treatment on these measures.	Sucrose (g/ml): 0.4, 02, 0.1, 0.05	NaCl (g/ml): 0.25, 0.1, 0.04, 0.016	Quinine Hydrochloride (g/ml): 0.006, 0.0024, 0.0009, 0.0004	N/A	Sour - Citric Acid (g/ml): 0.3, 0.165, 0.09, 0.05	Taste strips and taste identification	N/A	N/A	Whole mouth
Birmingham et al. (2005)	Cross-sectional	Psychophysical	To measure taste acuity of patients with Eating Disorders (Anorexia Nervosa, Bulimia Nervosa, and Eating Disorders Not Otherwise Specified) by identifying taste and describing	N/A	Standardized salt solution	Standardized bitter solution	Standardized umami solution	N/A	AccuSens Taste Kit - Taste acuity	N/A	N/A	Whole mouth

			strength of solution									
Blazer et al. (2008)	Cross-sectional	Psychophysical	To clarify whether taste and smell dysfunctions are specific or part of a pattern of sensory-perceptual deficits in Bulimia Nervosa patients.	Sucrose: 1%, 0.5%, and 0.025%	NaCl: 1%, 0.5%, and 0.025%	N/A	N/A	Citric Acid: 1%, 0.5%, and 0.025%; Urea: 1%, 0.5%, and 0.025%	Taste intensity and hedonic response	N/A	N/A	N/A
Bossert et al. (1991)	Pre/Post Treatment	Psychophysical	To see if bulimics and anorexics have higher palatability ratings to low calorie food images than controls	N/A	N/A	N/A	N/A	Viewing time of high calorie and low-calorie images	Taste preference	N/A	N/A	N/A
Dalton and Finlayson (2014)	Cross-sectional	Psychophysical	To measure implicit and explicit liking in relation to hedonics.	Photographs of high-fat sweets and low-fat sweets were presented	N/A	N/A	N/A	Photographs of savory foods that are low fat and high fat were presented	Leeds Food Preference Questionnaire	Fasted	Yes	N/A
Dazzi et al. (2013)	Cross-sectional	Psychophysical	To assess olfactory and gustatory function impairment in patients with eating disorders	Glucose (g/ml): 0.4, 0.2, 0.1 and 0.05	NaCl (g/ml): 0.25, 0.1, 0.04 and 0.016	Quinine Hydrochloride (g/ml): 0.006, 0.0024, 0.0009 and 0.0004	N/A	Citric Acid (g/ml): 0.3, 0.165, 0.09 and 0.05	Taste detection using taste strip kit	N/A	N/A	Anterior third of the right and left sides of the tongue
Drewnowski, Bellisle, et al. (1987)	Cross-sectional	Psychophysical	To assess taste responses of normal-weight bulimics with those of normal weight control women	Sucrose: 1%, 5%, 10%, 20%, and 40%	N/A	N/A	N/A	Fat (grams): 0, 3, 7 of fat per 100 grams of product	Taste and spit; hedonic response rated on 9-point scale	N/A	Participants were fed standard breakfast three hours before testing	Whole mouth
Drewnowski, Halmi, et al. (1987)	Pre/Post Treatment	Psychophysical	To examine the influence of body weight gain on taste responsiveness, anorectic patients were studied both at admission and following return to	Sucrose in each sample of dairy product: 0%, 5%, 10%, and 20%	N/A	N/A	N/A	Fat: skim milk- 0.1, milk- 3.5, half and half- 10.5, Heavy Cream-37.6, Cream and oil- 52.6 grams/100 grams	Taste and spit; hedonic response rated on 9-point scale	Overnight fast	N/A	Whole mouth

			target body weight"									
Drewnowski et al. (1992)	Pre/post Experimental	Psychophysical	To examine effects of opioids on taste preferences and food consumption patterns in compulsive binge eaters and in normal-weight female controls	Sucrose: 2%, 4%, 8%, 16%, and 32%	N/A	N/A	N/A	Fat: 3%, 10%, 20%, and 35% fat content	Food consumption; hedonic response	Overnight fast	N/A	Whole mouth
Drewnowski et al. (1995)	Experimental	Psychophysical	To test hypothesis that endogenous opiate peptides selectively influence hedonic response to sweet and high-fat foods, the opiate antagonist naloxone, opiate agonist butorphanol, and a saline placebo were administered by IV to 16 obese and 25 normal-weight women	Sucrose: 2%, 4%, 8%, 16%, and 32%	N/A	N/A	N/A	Fat: whole milk (3.5%), half and half (10% fat), light cream (20% fat), and heavy fat (36% fat)	Taste and spit; hedonic response rated on 9-point scale	Overnight fast	N/A	Whole mouth
Eiber et al. (2002)	Experimental	Psychophysical	To compare the hedonic responses to sucrose solutions in bulimics, restrictive anorexics and Anorectic-Bulimic women (ns20ygroup) in two different conditions: sucrose solution swallowed vs. sucrose solution spit.	Sucrose; 0%, 5%, 10%, 20%, and 40%	N/A	N/A	N/A	N/A	Solutions swallowed; solutions spit; Hedonic response and taste threshold	N/A	N/A	Whole mouth

Franko et al. (1994)	Cross-sectional	Psychophysical	To examine sweet taste intensity and pleasantness in Bulimia Nervosa patients	Sucrose: 0%, 1.3%, 2.6%, 5%, 10%, 20%, and 40%	N/A	N/A	N/A	N/A	Sip and spit; intensity and hedonic response	N/A	Participants consumed standardized breakfast prior to study	Whole mouth
Goldzak-Kunik et al. (2012)	Cross-sectional	Psychophysical	To examine sensory-perceptual function in a battery of tests that were designed to establish whether the pattern of sensory dysfunction reported in Anorexia Nervosa patients is indeed primarily related to eating, hunger, and body size or whether it is a general dysfunction.	Sucrose (mM): 3.2, 9.7, 29.2, 87.6, 262.9, 788.7	NaCl (mM): 2.5, 10, 40, 160, 640, 2560	Quinine Hydrochloride (mM): 0.01, 0.02, 0.04, 0.09, 0.18, 0.35	MSG (mmol/L): 0.6, 1.8, 5.9, 17.7, 53.2, 159.7	Citric Acid (mmol/L): 1,3,10,30, 93, and 280	Sip and spit; hedonic response	N/A	N/A	Whole mouth
Jirik-Babb and Katz (1988)	Cross-sectional	Psychophysical	To examine taste perception and intensity of sucrose, hydrochloride, NaCl, and quinine hydrochloride between controls and patients with Eating disorders (Anorexia Nervosa and Bulimia)	Sucrose (log M): -1.5, -1.0, -0.5, 0.0	NaCl (log M): -3.0, -2.0, -1.0, 0.0	Quinine Hydrochloride (log M): -5.0, -4.0, -3.0	N/A	HCl (log M): -3.0, -2.0, -1.5	Sip and spit; Taste identification	N/A	N/A	Whole mouth
Klein et al. (2010)	Cross-sectional	Psychophysical	To study taste preferences (namely orosensory stimulation) between bulimics and healthy women	Aspartame: 0%, 0.01%, 0.03%, 0.08%, 0.28%	N/A	N/A	N/A	N/A	Taste preference	N/A	Participants were fed ~300 kcal three to four hours before the experiment	Whole mouth

Lacey et al. (1977)	Cross-sectional	Psychophysical	To examine the sweetness taste sensitivity in patients with Anorexia Nervosa.	Sucrose: Five different concentrations were used but only 0.175% sucrose by weight was found to be in the region of uncertainty	N/A	N/A	N/A	N/A	Sip and spit; Taste sensitivity	Fasted	No	Whole mouth
Nakai et al. (1987)	Cross-sectional	Psychophysical	To examine the taste function in patients with Anorexia Nervosa and Bulimia Nervosa.	Sucrose: 0.3%, 2.5%, 10%, 20%, and 80%	NaCl: 0.3%, 1.25%, 5%, 10%, and 20%	Quinine: 0.001%, 0.02%, 0.1%, 0.5%, 4%	N/A	Sour - Tartrate: 0.02%, 0.2%, 2%, 4%, and 8%	Detection and recognition; filter paper disc method	N/A	N/A	Whole mouth
Nozoe et al. (1996)	Cross-sectional	Psychophysical	To monitor the taste sensitivity of nine Anorexia Nervosa patients on admission to hospital and during subsequent behavioral treatment.	Sucrose: 0.3%, 2.5%, 10%, 20%, 80%	NaCl: 0.3%, 1.25%, 5%, 10%, and 20%	Quinine: 0.001%, 0.02%, 0.1%, 0.5%, 4%	N/A	Sour - Tartrate: 0.02, 0.2, 2, 4, and 8%	Detection and recognition; filter paper disc method	N/A	N/A	Measurements were obtained from six sites on the right and left sides of the anterior and posterior lingual region, and soft palate
Peterson et al. (2016)	Quasi-experimental	Psychophysical	To compare profiles of hunger, fullness, olfactory, and gustatory responses in adolescent females (n = 15) with newly diagnosed restrictive eating disorders at hospital admission (i.e., severe malnutrition) and after medical refeeding, in comparison to healthy controls (n = 15)	N/A	N/A	N/A	N/A	N/A	Pleasant/unpleasant taste	N/A	N/A	N/A

Pierce et al. (1989)	Cross-sectional	Psychophysical	To examine the relationship between preference for sweetness or fat in a taste test and the patients' reported food preferences	Sucrose in each sample of dairy product: 0%, 5%, 10%, and 20%	N/A	N/A	N/A	Fat: 0.1%, 3.5%, 10.5%, 37.6%, 52.6%	Hedonic preference	N/A	N/A	N/A
Rodin et al. (1990)	Cross-sectional	Psychophysical	"To assess how the tongue and palate may be influenced by repeated contact with the acid in vomit, researchers in this study conducted specific spatial testing of various mouth regions. In order to determine whether disturbances in the perceived intensity or pleasantness of taste distinguish bulimics from normal subjects, subjects were asked to rate several solutions representing the four basic tastes—sweet, salty, sour and bitter"	Sucrose (M): 1, 0.32, 0.1, 0.032	NaCl (M): 1.0, 0.32, 0.1, 0.032	Quinine Hydrochloride (M): 0.001, 0.00032, 0.0001, 0.00032	N/A	Sour - Citric Acid (M): 0.001, 0.00032, 0.0001, 0.00032	Sip and spit; hedonic response	Overnight fast	N/A	Palate, back, front
Schebendach et al. (2014)	Cross-sectional	Psychophysical	To compare blinded ratings of the fat content of fat- free, low fat, and regular cream cheese in patients with Anorexia Nervosa and in normal controls.	N/A	N/A	N/A	N/A	Fat: Fat free, low fat, regular (0, 6, and 9 g fat per wt. oz. respectively)	Recognition thresholds	N/A	Yes; 2 hrs after lunch	Whole mouth
Simon et al. (1993)	Cross-sectional	Psychophysical	To see if taste preferences are an accurate index of body weight 'set	Sucrose: 1%, 5%, 10%, 20%, and 40%	N/A	N/A	N/A	Fat: soft white cheese containing 0, 3, and 7 grams of	Taste and spit; taste preference	Tested after overnight fast	Tested two hours after lunch	Whole mouth

			point'. Hypothesis is that anorectic women should find sucrose solutions acceptable even after a meal					fat per 100 grams				
S. R. Sunday and Halmi (1990)	Pre/Post Treatment	Psychophysical	To examine the taste hedonics and intensity ratings in anorectics of the restrictor-type (i.e., those who restrict their intake but do not binge), anorectic-bulimics, normal-weight bulimics, and normal-weight controls offered dairy solutions with varying amounts of fat and sucrose before and after treatment.	N/A	N/A	N/A	N/A	Twenty dairy solutions with four levels of sucrose and five levels of fat were used. The sucrose levels were 0%, 5%, 10%, and 20% wt./wt. The fat levels were 0.1%, 3.5%, 10.5%, 37.6%, and 52.6% wt./wt.	Taste preference	Overnight fast	No	Whole mouth
Suzanne R. Sunday and Halmi (1991)	Cross-sectional	Psychophysical	To examine the sensory and hedonic ratings of solutions that vary in fat and sucrose concentration in anorectic, anorectic-bulimic, bulimic, and control subjects. Subjects were tested before and after weight restoration for the anorectics and before and after cessation of binging and purging for the bulimics to assess whether sensory responses, preferences,	Sugar: 0%, 5%, 10%, 20%	N/A	N/A	N/A	Fat: 0.1%, 3.5%, 10.5%, 37.6%, 52.6%	Sip and spit; hedonic response	Fasted	N/A	Whole mouth

			and/or aversions are related to the current physiological status of the individual (e.g., body weight) or are stable, trait characteristics of eating disorders.									
Szalay et al. (2010)	Cross-sectional	Psychophysical	To clarify perceptual motivational aspects of gustatory disturbances in Anorexia Nervosa.	Sucrose (M): 0.1 and 0.5	NaCl (M): 0.1 and 0.5	Quinine Hydrochloride (mM): 0.3 and 3	MSG (M): 0.1 and 0.5	Complex (pleasant) taste from orange juice: 5% and 25%	Taste Reactivity; sip and spit	Fasted 6 hours	No	Whole mouth
						Self-Report						
Brand-Gothelf et al. (2016)	Cross-sectional	Self-report (Sensory Report Questionnaire)	To assess Sensory Modulation Disorder symptoms with Anorexia Nervosa Restrictors and Bulimia Nervosa in the acute phase of their illness, as well as in adolescent female patients with Anorexia Nervosa Restrictor following weight restoration	N/A	N/A	N/A	N/A	N/A	N/A	N/A	N/A	N/A
Koritar et al. (2017).	Cross-sectional	Self-report (Health and Taste Attitude Survey)	To establish if eating attitudes related to health concern or taste of food is different or similar in women with Bulimia Nervosa compared to a non-clinical sample and to test if body dissatisfaction is a	N/A	N/A	N/A	N/A	N/A	No taste test conducted; Health and Taste Attitude Scale used	N/A	N/A	N/A

			predictor of these attitudes									
Mitchell et al. (1999)	Cross-sectional	Self-report (Eating Hedonics Questionnaire)	To examine potential differences in the hedonics of binge eating between female subjects with Bulimia Nervosa (BN) and female subjects with Binge Eating Disorder (BED).	N/A	N/A	N/A	N/A	N/A	No Taste Test conducted; Eating Hedonics Questionnaire used. Looked at measures such as, "degree to which respondents endorsed pleasurable experiences".	N/A	N/A	N/A
Monje Moreno et al. (2014)	Cross-sectional	Thought-Shape Fusion Questionnaire	To explore the subjective reactivity after the exposure and tasting of foods with different gradient of sweetness and different fats textures. Thought-Shape fusion cognitive distortion was assessed.	Glucose (grams): 0, 40, 80	N/A	N/A	N/A	Fat: Bread with oil and tomato, bread with tomato mayonnaise and bread with olive oil and tomato meringue	Tasted sweets with different gradient of sweetness and fats with different textures	N/A	N/A	Whole mouth
Steinglass et al. (2015)	Cross-sectional	Visual Analog Scale (VAS)	To develop a new paradigm for experimentally modeling maladaptive food choice in Anorexia Nervosa. Participants viewed images of various foods on computer screen and indicated strength of preference for food item compared to neutral item; looked at FAT preferences. Also	N/A	N/A	N/A	N/A	N/A	Computer-based Food Choice Task.	N/A	N/A	N/A

			looked at "health" ratings relative to taste.									
						Imaging Studies						
Bohon and Stice (2012)	Cross-sectional	fMRI; psychophysical interaction analysis (for connectivity analysis)	"To evaluate the effect of negative affect on neural response to anticipation and receipt of palatable food in women with bulimia nervosa (BN) versus healthy controls. Also evaluated connectivity between the amygdala and reward-related brain regions."	N/A	N/A	N/A	N/A	N/A	No taste test conducted; tested anticipation and receipt of chocolate milkshake and tasteless solution	N/A	N/A	N/A
Frank et al. (2006)	Cross-sectional	fMRI	To determine whether bulimic women have alterations in the physiologic response to the blind administration of glucose.	Glucose (M): 1	N/A	N/A	N/A	Artificial saliva	Pump delivered taste stimuli during fMRI	Overnight	N/A	Whole mouth
Frank et al. (2011)	Cross-sectional	fMRI/psychometrics	To investigate DA-related responding in a classical conditioning paradigm.	Sucrose: 2%, 4%, 8%, 16%, 1 M	N/A	N/A	N/A	0% Distilled water; Artificial saliva (25mM KCl, 2 mM $NaHCO_3$)	Taste sensitivity	N/A	Meal plan breakfast; controls had breakfast matched for quality and calories	Whole mouth
Frank et al. (2012)	Cross-sectional	fMRI/psychometrics	To test whether there would be brain reward alterations in Anorexia Nervosa, compared with individuals with normal or increased body weight.	Sucrose: 2%, 4%, 8%, 16%, 1 M	N/A	N/A	N/A	0% Distilled water; artificial saliva (25mM KCl, 2 mM $NaHCO_3$)	Taste perception	N/A	Meal plan breakfast; controls had breakfast matched for quality and calories	Whole mouth

Frank, Shott, Hagman, et al. (2013)	Cross-Sectional	fMRI/psychometrics	Tested whether brain gray matter (GM) and white matter (WM) in adolescents with Anorexia Nervosa would show alterations comparable to adults.	Sucrose (M): 1	N/A	N/A	N/A	Control solution resembling saliva	N/A	N/A	Meal plan	N/A
Frank, Shott, and Mittal (2013)	Cross-sectional	fMRI/psychometrics	To study gray and white matter volumes in individuals with restricting type currently ill Anorexia Nervosa, recovered Anorexia Nervosa, Bulimia Nervosa, and healthy control women	Sucrose: 2%, 4%, 8%, 16%, 1 M	N/A	N/A	N/A	0% Distilled water; Artificial saliva (25mM KCl, 2 mM NaHCO3)	Taste perception	N/A	Meal plan breakfast; controls had breakfast matched for quality and calories	Whole mouth
Frank, Collier, et al. (2016)	Cross-sectional	fMRI/psychometrics	To identify whether higher prediction error brain reward response or sensitivity using the same paradigm is also present in individuals recovered from anorexia nervosa.	Sucrose: 2%, 4%, 8%,16%, 1M	N/A	N/A	N/A	Water, Artificial saliva (25 mM of potassium chloride, 2mM of sodium bicarbonate)	Pump delivered taste stimuli during fMRI	N/A	Standardized breakfast	Whole mouth
Frank, Shott, Riederer, et al. (2016)	Cross-sectional	fMRI/psychometrics	To use a multimodal imaging approach to test the hypothesis that individuals ill with anorexia and bulimia nervosa have greater white matter connectivity across the energy homeostasis and cognitive-	Sucrose (M): 1	N/A	N/A	N/A	No solution, Artificial saliva (also included a conditioned visual stimulus for each solution)	Pump delivered taste stimuli during fMRI	N/A	Standardized breakfast	Whole mouth

			emotional reward circuitry.									
Frank, Shott, Keffler, et al. (2016)	Cross-sectional	fMRI/psychometrics	to 1) determine the best model of pattern activation in this region across participants and 2) compare taste classification pattern accuracy between groups, while correcting for potential confounds such as comorbidity, medication use, brain volume, but also interoceptive or taste perception differences, factors that may fluctuate with anxiety and self-restraint.	Sucrose: 2%, 4%, 8%, 16%, 1 M	N/A	N/A	N/A	Distilled water; Artificial saliva (25mM KCl, 2mM NaHCO3)	Taste sensitivity	Taste stimuli- before breakfast	Meal plan breakfast; controls had breakfast matched for quality and calories	Whole mouth
Monteleone et al. (2017)	Cross-sectional	fMRI/psychometrics	To investigate and contrast the representations of a pleasant (sucrose solution) and an aversive (quinine hydrochloride solution) basic taste in the brain of symptomatic patients with Anorexia Nervosa and Bulimia Nervosa compared to healthy subjects.	Sucrose (M): 0.292	N/A	Quinine Hydrochloride (mM): 0.5	N/A	Water	Pump delivered taste stimuli during fMRI	6 hours	N/A	Whole mouth
Oberndorfer et al. (2013)	Cross-sectional	fMRI/self-report	To use sweet tastes to interrogate gustatory neurocircuitry involving the	Sucrose; sucralose	N/A	N/A	N/A	N/A	Pump delivered taste stimuli during fMRI	N/A	Standardized breakfast	Whole mouth

			anterior insula and related regions that modulate sensory-interoceptive-reward signals in response to palatable foods.									
Radeloff et al. (2014)	Cross-sectional	fMRI/self-report	To investigate neuronal pathways that may contribute to altered fat consumption in eating disordered patients.	N/A	N/A	N/A	N/A	High-fat whipping cream (0.1, 3, 10, 30%); Tasteless thickening agent dissolved in water and sweetened to be comparable to whipping cream; water	Cue and tube delivery of taste stimuli during fMRI	4 hours after standardized breakfast	N/A	Whole mouth
Toth, Kondakor, et al. (2004)	Cross-sectional	EEG	To investigate the effects of pleasant and unpleasant gustatory stimuli on nonlinear and linear complexity measures of the EEG in healthy controls and in anorexia nervosa patients.	Pleasant (chocolate milk): 4.0 grams	N/A	Unpleasant (bitter): 50 ml	N/A	N/A	EEG and tastant	N/A	N/A	Whole mouth
Toth, Tury, et al. (2004)	Cross-sectional	EEG	To study was to investigate the effects of pleasant (sweet) and unpleasant (bitter) stimuli on the EEG recorded in healthy persons and Anorexia Nervosa patients in order to elucidate the possibly altered processing mechanisms of these stimuli in Anorexia Nervosa.	Milk Chocolate	N/A	Black Tea	N/A	N/A	EEG and tastant	N/A	N/A	Whole mouth

Vocks et al. (2011)	Cross-sectional	fMRI/self-report	To examine possible alterations in neuronal correlates of gustatory processing of food stimuli in Anorexia Nervosa and to test the impact of hunger and satiety.	N/A	N/A	N/A	N/A	Chocolate milk (1.5% fat); water	Water vs chocolate milk in hungry vs satiated conditions	N/A	Standardized breakfast of 2 slices of toast with butter and jam to be "slightly hungry"; bread rolls with butter and cheese, water until full	Whole mouth
Wagner et al. (2008)	Cross-sectional	fMRI/self-report	To determine whether individuals recovered from Anorexia Nervosa have an abnormal physiological response to sugar or water.	Sucrose: 10%	N/A	N/A	N/A	Distilled water	Pump delivered taste stimuli during fMRI	N/A	Standardized breakfast	Whole mouth
Wagner et al. (2015)	Cross-sectional	fMRI	To determine whether sensitization effects might underlie pathologic eating behavior when a taste stimulus is administered repeatedly.	Sucrose: 10%; sucralose: 10%	N/A	N/A	N/A	N/A	Pump delivered taste stimuli during fMRI	N/A	Standardized breakfast	Whole mouth

Table S3. Study results regarding taste differences among patients with eating disorders and controls

Anorexia Nervosa							
**Citation**	**Taste(s)**	**Preference**	**Pleasantness and Palatability**	**Detection (Sensitivity and Intensity)**	**Taste Questionnaire**	**Brain Sensory Responsiveness**	**Gustatory Function**
Frank et al. (2012)	Sweet	AN=controls=OB	AN=controls=OB regarding pleasantness	AN=controls=OB	DNM	DNM	DNM
Frank, Shott, Hagman, et al. (2013)	Sweet	DNM	AN < controls regarding orbitofrontal gray matter volume and pleasantness	AN=controls regarding perception	DNM	DNM	DNM
Frank, Shott, Keffler, et al. (2016)	Sweet	AN=controls	AN=controls regarding pleasantness	DNM	DNM	DNM	DNM
Goldzak-Kunik et al. (2012)	DNM	DNM	DNM	AN = controls regarding sensitivity	DNM	AN had a better response to odor recognition, but less sensitive to auditory and oral stimuli	No difference between AN and controls
Lacey et al. (1977)	Sweet	DNM	DNM	No difference in sucrose sensitivity between AN and controls. Taste sensitivity related to caloric intake in both AN and controls	DNM	DNM	DNM
Monje Moreno et al. (2014)	Sweet and fat	AN > controls especially when a less gradient of sweetness was present	DNM	DNM	TSF-Questionnaire – AN > controls (sweet). AN < controls (pre-fats testing)	DNM	DNM
Nozoe et al. (1996)	DNM	DNM	DNM	DNM	DNM	AN < controls when admitted, but slightly increased during treatment	DNM
Schebendach et al. (2014)	Fat	DNM	DNM	DNM	DNM	DNM	No difference between AN and controls
Simon et al. (1993)	Sweet and fat	Preference for sweet did not differ between AN and controls<br>AN < controls (fat)	DNM	AN=controls (sweet) regarding perception	DNM	DNM	DNM

Steinglass et al. (2015)	Fat	AN preferred low-fat food	DNM	DNM	Computer-based Food Choice Task	DNM	DNM
Szalay et al. (2010)	Pleasantness	DNM	AN < controls regarding pleasantness	DNM	DNM	DNM	DNM
Toth, Kondakor, et al. (2004)	Pleasantness and bitter	DNM	DNM	DNM	DNM	AN < controls regarding dimensional complexity	DNM
Toth, Tury, et al. (2004)	Sweet and bitter	DNM	DNM	DNM	DNM	Pattern of activation in AN differed from controls	DNM
Vocks et al. (2011)	DNM	DNM	DNM	DNM	DNM	Differences in activation of the amygdala, insula, and temporal gyrus were seen between AN and controls during hunger and satiety	DNM
Bulimia Nervosa							

Blazer et al. (2008)	Salt, sweet, sour, and bitter	DNM	BN<control (salt); BN=control (sweet); BN=control (sour); BN=control (bitter) regarding pleasantness	BN<control (salt); BN=control (sweet); BN=control (sour); BN=control (bitter) regarding perception	BN-related questionnaires	DNM	DNM
Bohon and Stice (2012)	DNM	DNM	DNM	DNM	DNM	BN > controls regarding amygdala activation	BN < controls
Drewnowski, Bellisle, et al. (1987)	Sweet and fat	BN > control regarding optimal sweetness	DNM	BN=control regarding perception	DNM	DNM	DNM
Franko et al. (1994)	Sweet	DNM	DNM	BN=control regarding intensity	DNM	DNM	DNM
Frank et al. (2011)	Sweet	BN=controls	BN=controls regarding pleasantness	BN > controls regarding sensitivity to reward and punishment	Sensitivity to Reward and Punishment Questionnaire	BN < control	DNM
Klein et al. (2010)	Sweet	BN=controls	DNM	Participants with BN had 40.5-53.1% greater intake than controls	DNM	BN=controls	DNM

Koritar et al. (2017).	DNM	DNM	Controls > BN regarding pleasantness	Participants with BN had higher reference for “light” products	Health and Taste Attitude Scale. Controls > BN (Taste Domain) BN > controls (Health Domain)	DNM	DNM
Pierce et al. (1989)	Sweet and fat	BN < controls when high fat diet was not masked by sweetness. AN < controls regarding low carb/high fat and high carb/low fat diets	DNM	No difference between BN, AN, and controls regarding intensity for sweet and fat	DNM	DNM	DNM
Rodin et al. (1990)	Sweet, salty, bitter, and sour	BN < controls (sweet)	No difference between BN and controls regarding pleasantness	No difference between BN and controls regarding intensity	DNM	DNM	DNM
Binge Eating Disorder							
Arlt et al. (2017)	Sweet and bitter	DNM	DNM	Overweight BED < controls and normal-weight BED regarding perception	DNM	DNM	DNM
Dalton and Finlayson (2014)	Sweet and fat	BED > controls regarding preference and wanting of high-fat sweet foods	DNM	DNM	Binge Eating Scale. High-scorer’s intake > low-scorer’s intake	DNM	DNM
Drewnowski et al. (1992)	Sweet and fat	Preference decreased in BED and controls due to Naloxone	DNM	DNM	DNM	DNM	DNM
Drewnowski et al. (1995)	Sweet and fat	Naloxone caused suppression of sweet and high-fat foods only in BED	DNM	DNM	DNM	DNM	DNM
Multiple Eating Disorders							
Aschenbrenner et al. (2008)	DNM	DNM	DNM	DNM	DNM	DNM	Controls > BN > AN. Improved over time in AN
Bossert et al. (1991)	DNM	AN did not prefer high-caloric foods	BN and AN < controls regrading palatability	DNM	DNM	DNM	DNM

Brand-Gothelf et al. (2016)	DNM	DNM	DNM	DNM	SRQ Taste/Gustatory – AN > BN + controls	AN (ill and weight restored) > BN + controls	DNM
Dazzi et al. (2013)	Bitter	DNM	DNM	AN and BN < control (bitter) regarding perception	DNM	DNM	AN and BN < controls
Drewnowski, Halmi, et al. (1987)	Sweet and fat	BN > control regarding sweet preference<br>AN and BN < controls regarding high-fat	AN=BN=control regarding pleasantness	AN=BN=control regarding perception	DNM	DNM	DNM
Eiber et al. (2002)	Sweet	Taste hedonics was dependent on whether participants with AN, RAN, or BN swallowed or spit solutions	DNM	DNM	DNM	DNM	DNM
Frank, Shott, Riederer, et al. (2016)	Sweet	DNM	AN=BN=control regarding pleasantness	AN=BN=control regarding perception	DNM	DNM	DNM
Jirik-Babb and Katz (1988)	Sweet, salt, and bitter	DNM	DNM	Magnitude of AN and BN < controls. Gustatory sensitivity associated with abnormal eating behavior in AN and BN	DNM	DNM	DNM
Mitchell et al. (1999)	DNM	DNM	BED < BN regarding physical discomfort while consuming foods. BED > BN regarding food enjoyment and taste	DNM	Eating Hedonics Questionnaire	DNM	DNM
Monteleone et al. (2017)	Sweet and bitter	DNM	AN=BN=control regarding pleasantness	AN=BN=control	DNM	AN and BN < controls regarding bitter taste	DNM

Nakai et al. (1987)	Sweet, salt, and bitter	DNM	DNM	DNM	DNM	DNM	Taste function increased in AN and BN
Radeloff et al. (2014)	Fat	DNM	DNM	DNM	DNM	BN > AN and controls regarding magnitude of response	DNM
S. R. Sunday and Halmi (1990)	Sweet and fat	DNM	DNM	BN > AN and controls during treatment regarding ratings of low-fat and sugar-free solutions	DNM	DNM	DNM
Suzanne R. Sunday and Halmi (1991)	Sweet and fat	AN differed from controls in hedonic ratings	DNM	DNM	DNM	No difference between AN, BN, and controls regarding sweet and fat taste	DNM
Eating Disorder Not Specified							
Peterson et al. (2016)	DNM	DNM	Participants with ED < controls regarding pleasantness	DNM	DNM	DNM	DNM
Recovered Anorexia Nervosa							
Frank, Collier, et al. (2016)	Sweet	RAN=controls	RAN=controls regarding pleasantness	DNM	DNM	DNM	DNM
Wagner et al. (2008)	DNM	DNM	DNM	DNM	DNM	AN < controls regarding activation of insula	DNM
Recovered Bulimia Nervosa							
Frank et al. (2006)	Sweet	DNM	DNM	DNM	DNM	RBN < control regarding activation of anterior cingulate cortex	DNM
Remitted/Recovered Multiple Eating Disorders							
Frank, Shott, and Mittal (2013)	Sweet	AN > RAN	AN=BN=control regrading pleasantness	DNM	DNM	DNM	DNM
Frank, Shott, Keffler, et al. (2016)	Sweet	AN=BN=OB=RAN=control s	AN=BN=OB=RAN=controls regarding pleasantness	DNM	DNM	DNM	DNM
Oberndorfer et al. (2013)	DNM	DNM	DNM	DNM	DNM	RAN < RBN regarding sweet taste	DNM
Radeloff et al. (2014)	Fat	DNM	DNM	DNM	DNM	RBN > RAN and controls regarding magnitude of response	DNM
Wagner et al. (2015)	Sweet	DNM	DNM	DNM	DNM	RAN < RBN regarding sensitivity	DNM

Legend: AN = Anorexia Nervosa; BN = Bulimia Nervosa; BED = Binge Eating Disorder; OB = Obesity; DNM = Did not measure; RAN = Remitted Anorexia Nervosa; RBN = Remitted Bulimia Nervosa; Gustatory Function = Studies that did measure taste preference and detection, but accounted for taste function
